# *N-*Propargylamines: versatile building blocks in the construction of thiazole cores

**DOI:** 10.3762/bjoc.13.61

**Published:** 2017-03-30

**Authors:** S Arshadi, E Vessally, L Edjlali, R Hosseinzadeh-Khanmiri, E Ghorbani-Kalhor

**Affiliations:** 1 Department of Chemistry, Payame Noor University, Tehran, Iran; 2 Department of Chemistry, Tabriz Branch, Islamic Azad University, Tabriz, Iran

**Keywords:** 6-*endo-dig* cyclization, 5-*exo-dig* cyclization, *N*-heterocycles, *N*-propargylamines, thiazoles

## Abstract

Thiazoles and their hydrogenated analogues are not only key structural units in a wide variety of natural products but they also constitute important building blocks in medicinal chemistry. Therefore, the synthesis of these compounds using new protocols is always interesting. It is well known that *N*-propargylamines can undergo a number of cyclization reactions to produce various nitrogen-containing heterocycles. In this review, we highlight the most important developments on the synthesis of thiazole and its derivatives starting from *N*-propargylamines. This review will be helpful in the development of improved methods for the synthesis of natural and biologically important compounds.

## Introduction

**Figure 1:**
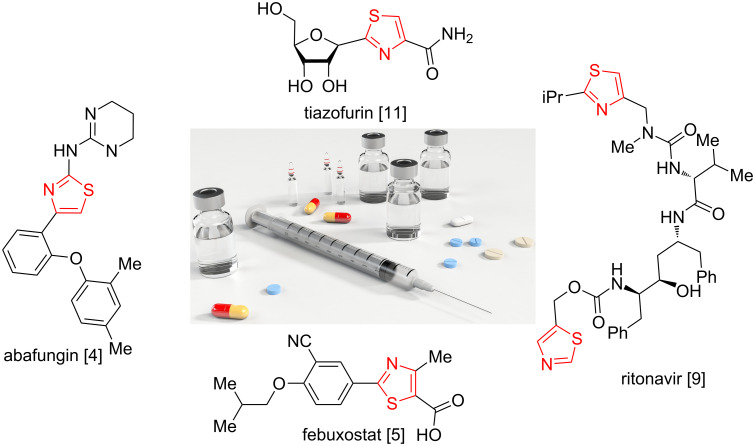
Selected examples of bioactive thiazole derivatives.

**Figure 2:**
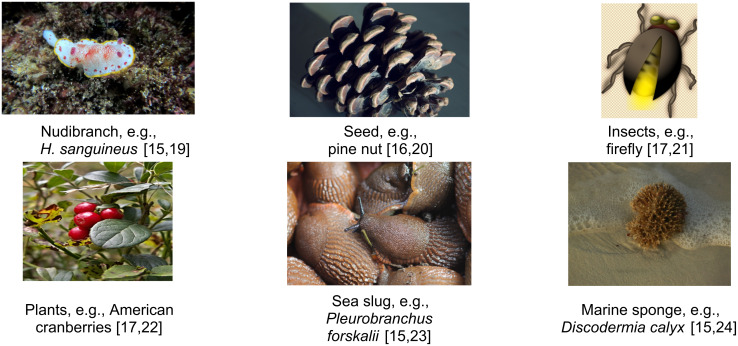
Some natural sources of thiazoles.

Thiazoles are an important class of azole compounds that have attracted considerable attention due to the fact that they exhibit a wide variety of pharmacological activities. For example, abafungin (Figure 1) is an antifungal drug marketed worldwide for the treatment of dermatomycoses. It works by inhibiting the enzyme sterol 24-C-methyltransferase [1–4]. Febuxostat, also known by its brand name adenuric is a xanthine oxidase inhibitor that helps to prevent gout flare-ups [5–7]. Ritonavir (norvir), is an HIV protease inhibitor. It works by blocking the growth of HIV [8–9]. Tiazofurin is a C-nucleoside analogue with antineoplastic activity and acts by inhibition of the guanosine triphosphate (GTP) biosynthesis through a reduction of PI and PIP kinase activity [10–14] (Figure 1). This compound class is also a crucial part of many natural products such as vitamin B1 (thiamine), epothilone, dolastatin, and many more (Figure 2) [15–24]. Moreover, thiazoles are widely applied as pesticides and dyes [25]. As a consequence, many routes for the synthesis of thiazole derivatives are reported in the literature [26–33]. Among them, the Hantzsch thiazole synthesis (condensation of α-haloketones with thioamides) is the most efficient and straight forward procedure [34–43]. However, the general applicability of this method is limited by the narrow substitution patterns [31], by the harsh reaction conditions [26,30] or both. Therefore, methods that overcome these drawbacks are required.

The hydrogenated thiazoles (thiazoline and thiazolidine derivatives) are also important structural motifs that are widely found in biologically active natural or synthetic products [44–50]. Compounds containing these rings have widespread biological applications as anticancer [51–53], anti-HIV [54–55], anti-inflammatory [56], antimicrobial [57–59], and specially antibiotic [60–64] agents. Despite their great relevance in drug design, only very few synthetic methods towards these compounds have been reported to date [44].

**Figure 3:**
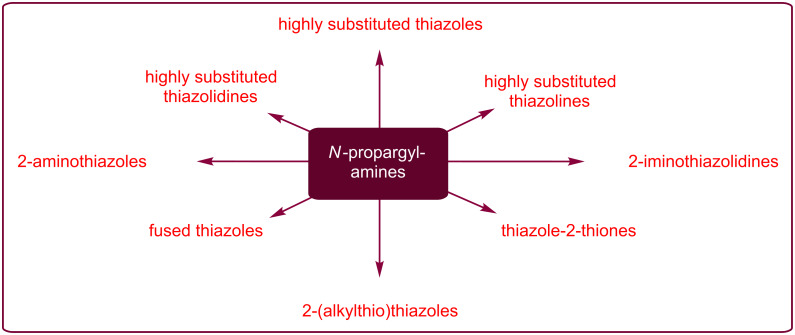
Some important thiazole-based compounds derived from *N*-propargylamines.

*N*-Propargylamines are one of the most specific class of alkynes having diverse reaction patterns. It is well known that they can undergo a number of cyclization reactions to produce various *N*-heterocycles and complex natural products. In this context we recently reviewed their role in the syntheses of pyrrole [65], pyridine [66], quinoline [67], pyrazine [68], 1,4-oxazepane, and 1,4-diazepane [69] derivatives. The synthesis of thiazoles and their hydrogenated analogues from *N*-propargylamines offers several advantages, such as high functional group tolerance and high atom and step economy. In continuation of our works [69–74], in this review, we will highlight the most important developments on the synthesis of thiazole and its derivatives from *N*-propargylamines (Figure 3) which will be helpful in the development of improved methods for the synthesis of natural and biologically important compounds. The review is organized by the type of starting materials.

## Review

### 1 From *N*-propargylamines and carbon disulfide

**Scheme 1:**
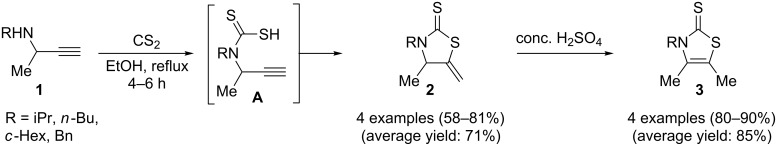
The synthesis of thiazole-2-thiones **3** through the thermal cyclocondensation of *N*-propargylamines **1** with carbon disulfide as developed by Batty and Weedon [75].

The first example of a cyclization of *N*-propargylamines **1** with carbon disulfide to lead to 5-methylenethiazolidine-2-thiones **2** was reported in 1949 by Batty and Weedon. The reaction took place in refluxing ethanol and generally afforded the corresponding products in good yields. It was also observed that products **2** rapidly formed by reaction of **1** with carbon disulfide in the presence of sodium hydroxide as the base in water at 20 °C. Further, the authors showed that treatment of methylene compound **2** with cold concentrated sulfuric acid gave the corresponding isomeric thiazole-2-thiones **3** in high yields (Scheme 1) [75]. Thirty-six years later, Hanefeld and Bercin synthesized a series of 2-(alkylthio)thiazoles by employing the aforementioned method as the key step [76]. In 2001, Shi and Shen found that using a Pd(PPh_3_)_4_/toluene system clearly accelerated this cyclocondensation and the desired products were obtained in excellent yields [77]. Other systems such as Pd(OAc)_2_/THF [78], D301R (a tertiary amine-functionalized ion-exchange resin)/biphenyl [79], and diethylamine/NaOH/H_2_O [80] were also successfully employed in this transformation. Despite all these successes, the number of reported examples in this interesting field is limited. There is still further need to study the scope and limitations of this approach for the preparation of thiazolidine-2-thione derivatives.

**Scheme 2:**
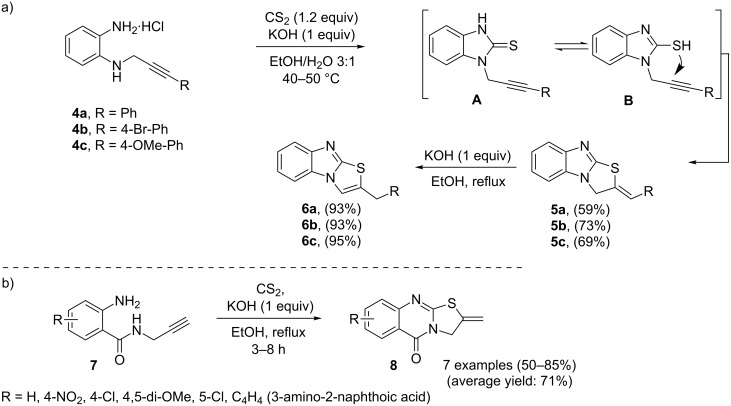
(a) One-pot synthesis of 2-benzylthiazolo[3,2-*a*]benzimidazoles **6** through a base-catalyzed cascade reaction of internal *N*-propargylamines **4** and CS_2_. (b) Synthesis of 2-methylene-thiazolo[2,3-*b*]quinazolinones **8** using 2-amino-propargylbenzamides **7** as substrates.

A straightforward way towards 2-benzylthiazolo[3,2-*a*]benzimidazole derivatives **6** has been proposed by Balova et al. In their approach, a sequential cyclocondensation/5-*exo-dig* cyclization process between 2-amino-*N*-propargylanilines **4** and CS_2_ afforded heterocyclic systems of type **5**. Isomerization of the latter compounds upon heating in the presence of KOH in ethanol gave the corresponding 2-benzylthiazolo[3,2-*a*]benzimidazoles **6** in good yields (Scheme 2a) [81]. In a closely related investigation, Shafiee and co-workers also found that the cyclocondensation of 2-amino-*N*-propargylbenzamides **7** with CS_2_ in a KOH/EtOH system gave the corresponding 2-methylenethiazolo[2,3-*b*]quinazolinones **8** in good to high yields (Scheme 2b) [82].

### 2 From *N*-propargylamines and isothiocyanates

**Scheme 3:**
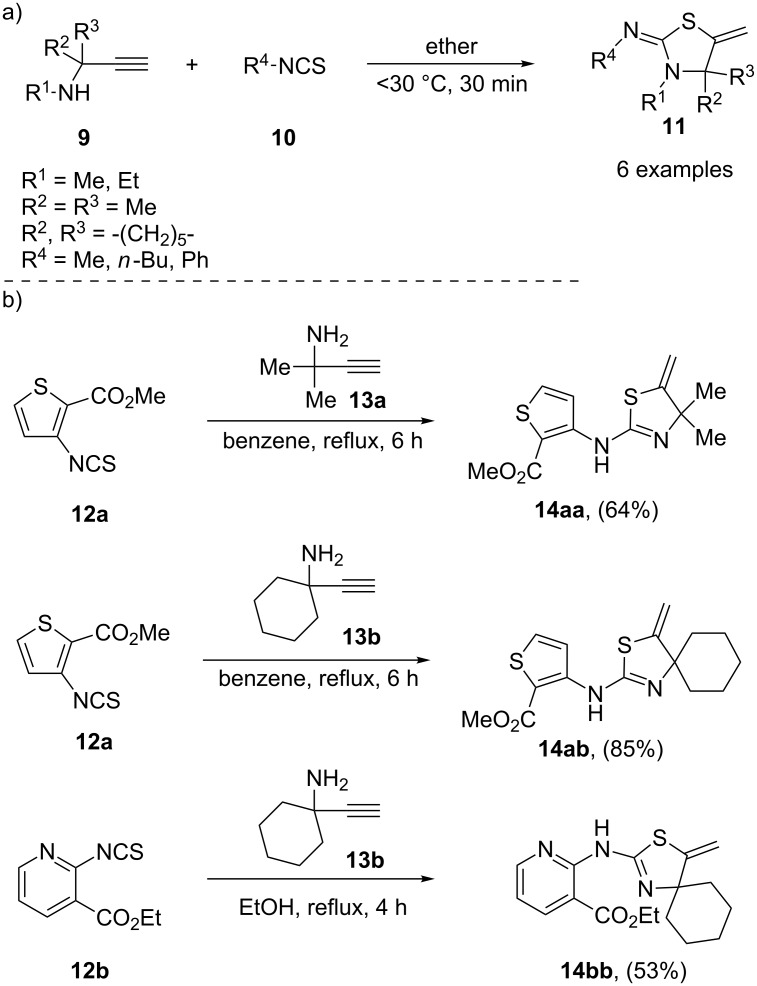
(a) Synthesis of 2-iminothiazolidines **11** from *N*-propargylamines **9** and isothiocyanates **10**. (b) Synthesis of 4,4-disubstituted-5-methylenethiazoles **14** using heterocyclic isothiocyanates **12** and α,α-disubstituted *N*-propargylamines **13** as substrates.

The first example of a synthesis of thiazole derivatives from *N*-propargylamines and isothiocyanates was reported in 1964 by Easton et al. The authors obtained 2-iminothiazolidines **11** in good yields by the treatment of secondary α,α-disubstituted *N*-propargylamines **9** with isothiocyanates **10** through a catalyst-free thiourea formation/intramolecular thia-Michael cyclization in ether (Scheme 3a). They also showed that the treatment of primary α,α-disubstituted *N*-propargylamines with isothiocyanates led to the corresponding *N*-propargylthioureas that converted to the cyclic forms upon standing for several days at room temperature [83]. Thirteen years later, Arya and co-workers applied this method for the synthesis of 3-thia-1-azaspiro[4,5]decane ring systems [84]. In 1993, U. Urleb extended the scope of the reaction from isothiocyanates to heterocyclic isothiocyanates and some reported examples are shown in Scheme 3b [85].

**Scheme 4:**
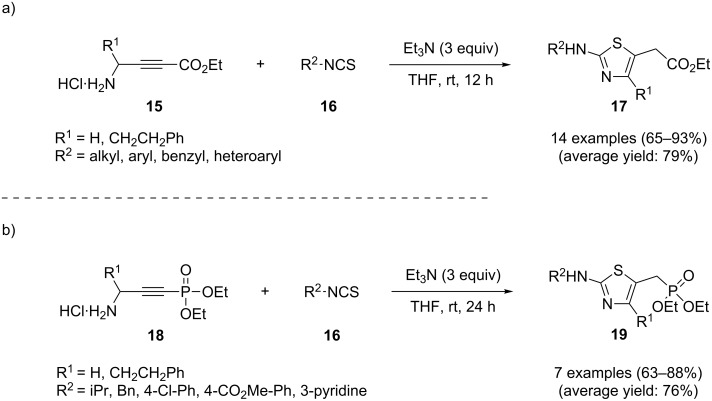
(a) Synthesis of 2-aminothiazoles **17** through the reaction of ethyl 4-aminobut-2-ynoate salts **15** with isothiocyanates **16.** (b) Synthesis of 5-diethyl methylphosphonate-substituted 2-aminothiazoles **19** through reaction of diethyl 3-aminoprop-1-ynylphosphonate salts **18** with **16**.

This strategy was elegantly used by Sasmal and co-workers in the preparation of 2-aminothiazoles **17** from ethyl 4-aminobut-2-ynoate salts **15** and isothiocyanates **16**. Several bases and solvents were screened and the combination of Et_3_N and THF at room temperature was found to be superior. Under the optimized conditions, the reaction tolerates both aryl and alkyl isothiocyanates **16** and gave the corresponding 2-aminothiazoles **17** in good to high yields (Scheme 4a). The authors further expanded the scope of *N*-propargylamines to diethyl 3-aminoprop-1-ynylphosphonate salts **18** leading to 5-diethyl methylphosphonate-substituted 2-aminothiazoles **19** in good yields (Scheme 4b) [86].

An interesting approach towards the synthesis of 2-aminothiazole derivatives by treatment of *N*-propargylamines with isothiocyanates in the presence of *p*-toluenesulfonic acid (PTSA) as catalyst under microwave irradiation was developed by Castagnolo et al. Following this route, several 4-substituted 5-methylthiazol-2-amines **22** were synthesized from terminal *N*-propargylamines **20** and isothiocyanates **21** in DMF at 160 °C. Interestingly, when internal *N*-propargylamines were treated with **21**, exclusively imidazolthiones **24** in yields ranging from 15 to 33% instead of the expected 2-aminothiazoles were obtained. The authors also found that with decreasing reaction temperature the yield of **22** decreased in favor of the thiazolines **23**. Some reported examples are collected in Table 1 [87].

**Table 1:** Microwave-assisted domino reactions of *N*-propargylamines **20** with isothiocyanates **21** developed by Castagnolo.

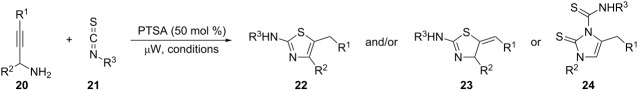								

Entry	R^1^	R^2^	R^3^	Solvent	Temp (°C)	Product	Ratio **22**:**23**:**24**	Yield (%)

1	H	Ph	All	DMF	160	**3a**	100:0:0	47
2	H	Ph	Bn	DMF	160	**3b**	100:0:0	56
3	H	4-Cl-Ph	All	DMF	160	**3c**	100:0:0	55
4	H	2,4-Cl_2_-Ph	Bn	DMF	160	**3d**	100:0:0	62
5	H	2,4-Cl_2_-Ph	All	DMF	160	**3e**	100:0:0	62
6	Ph	H	Bn	DMF	160	**5a**	0:0:100	15
7	Ph	H	All	DMF	160	**5b**	0:0:100	33
8	Ph	H	Ph	DMF	160	**5c**	0:0:100	21
9	H	H	Bn	MeCN	100	**3f**/**4a**	25:75:0	18:60
10	H	H	Ph	MeCN	100	**3g**/**4b**	20:80:0	12:57
12	H	2,4-Cl_2_-Ph	All	DCE	130	**4c**	0:100:0	60

**Scheme 5:**
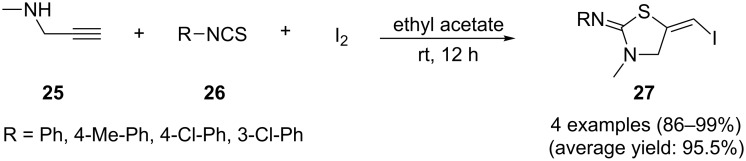
Synthesis of 5-(iodomethylene)-3-methylthiazolidines **27** described by Zhou.

**Scheme 6:**
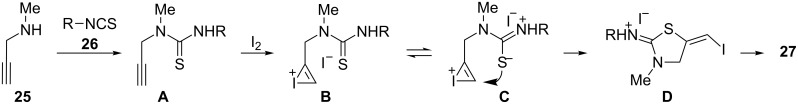
Mechanism that accounts for the formation of **27**.

Recently, to develop an efficient protocol for the synthesis of 5-(iodomethylene)-3-methylthiazolidines **27** from *N*-propargylamines, X. Zhou and co-workers have investigated the three-component halocyclization of *N*-propargylamines **25**, aryl isothiocyanates **26**, and iodine in ethyl acetate. Excellent yields of desired products were observed (Scheme 5). The mechanism shown in Scheme 6 was proposed for this transformation and comprises the following key steps: (i) the reaction of *N*-propargylamine **25** and isothiocyanate **26** forms the thiourea intermediate **A**, (ii) electrophilic addition of I_2_ to the alkyne moiety of this intermediate produces the iodonium intermediate **B**, (iii) isomerization of iodonium **B** gives intermediate **C** and (iv) a sequential intramolecular cyclization and HI elimination of C finally affords thiazolidines **27** [88].

**Scheme 7:**
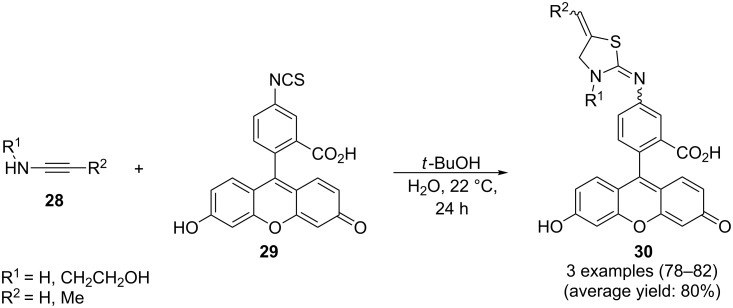
Clausen’s synthesis of fluorescein thiazolidines **30**.

Seeking for a greener approach towards thiazolidines of type **30**, the group of Clausen has proposed a base-catalyzed protocol using *t*-BuOH in water at 20 °C for a quite efficient cyclization between secondary *N*-propargylamines **28** and fluorescein isothiocyanate **29** (Scheme 7) [89].

**Scheme 8:**
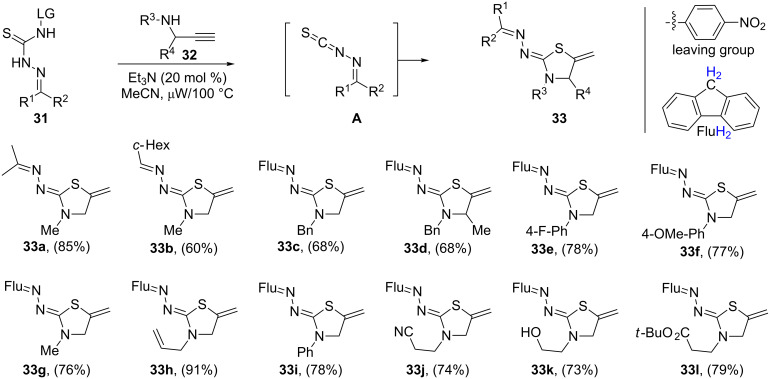
Synthesis of multiply substituted thiazolidines **33** from *N*-propargylamines **32** and blocked *N*-isothiocyanate precursors **31**.

More recently, Beauchemin and co-workers reported the syntheses of a series of multiply substituted thiazolidines **33** via the cyclization reaction of secondary *N*-propargylamines **32** with blocked *N*-isothiocyanate precursors **31**. The desired *N*-isocyanates **A** are produced in situ upon heating or treatment with a base, in acetonitrile under microwave irradiation conditions (Scheme 8). The reaction tolerated a variety of functional groups such as fluoro, cyano, hydroxy, and methoxy, allowing a further derivatization of the products [90].

### 3 From *N*-propargyl thioamides

**Scheme 9:**
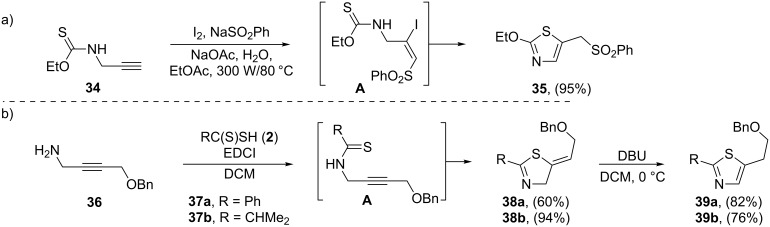
(a) Microwave-assisted cyclization of *N*-propargyl thiocarbamate **34**. (b) Synthesis of thiazoles **39** through a tandem coupling–cyclization–isomerization sequential process.

The first example of a thiazole synthesis from *N*-propargyl thioamides has been reported by Short and Ziegler in 1993. *N*-Propargyl thiocarbamate **34** cyclized to disubstituted thiazole **35** through an addition–cycloelimination strategy by the treatment with sodium benzenesulfinate and I_2_ in ethyl acetate and water at 80 °C (Scheme 9a) [91]. Later, the P. Wipf research team found that *N*-propargylamines **36** were converted to the corresponding vinylthiazolines **38** through the treatment with dithioic acids **37** in the presence of EDCI in dichloromethane. This transformation is believed to occur through a tandem coupling–cyclization reaction. The authors showed that the treatment of **38** with DBU at 0 °C provided thiazoles **39** in good yields (Scheme 9b) [92].

**Scheme 10:**
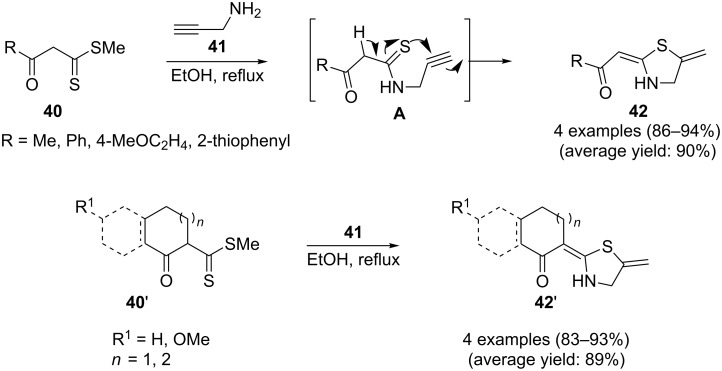
Synthesis of thiazolidines **42** (**42’**) from the reaction of β-oxodithioesters **40** (**40’**) with *N*-propargylamine (**41**) through an *N*-propargylthioamide intermediate **A**.

Along this line, Junjappa and co-workers reported an efficient route for the synthesis of 2-substituted 5-methylenethiazolidines **42** through the reaction of β-oxodithioesters **40** with *N*-propargylamine (**41**). The mechanism proposed by the authors to explain this reaction is based on the formation of β-oxo-*N*-propargyl thioamides **A** as intermediates, followed by their spontaneous ring closure. This reaction was run in refluxing ethanol and provided in all cases the desired thiazolidines **42** in high to excellent yields (Scheme 10) [93].

**Scheme 11:**
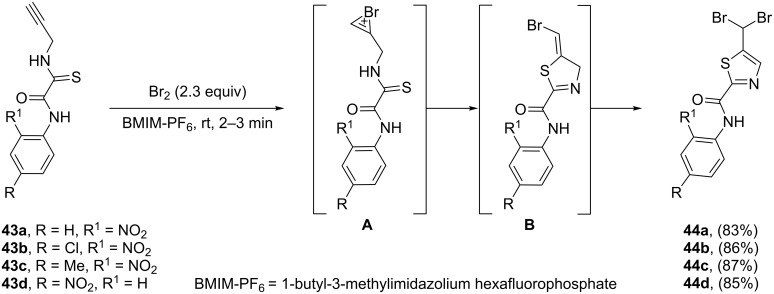
Synthesis of 5-(dibromomethyl)thiazoles **44** via halocyclization of *N*-propargylamines **43** described by Yarovenko.

In 2009, Yarovenko and co-workers developed the synthesis of 5-(dibromomethyl)thiazole derivatives **44** by treatment of *N*-propargyl thioamides **43** with bromine in an ionic liquid (1-butyl-3-methylimidazolium hexafluorophosphate). Mechanistically, the reaction involves: i) bromination of triple bond of thioamide **43** which resulted in a bridged bromonium ion intermediate **A**; ii) regioselective 5-*exo-dig* cyclization of intermediate **A** to give dihydrothiazole **B**; and iii) addition of a second bromine to the alkene moiety in intermediate **B** to provide the corresponding thiazole **44** (Scheme 11) [94].

**Scheme 12:**
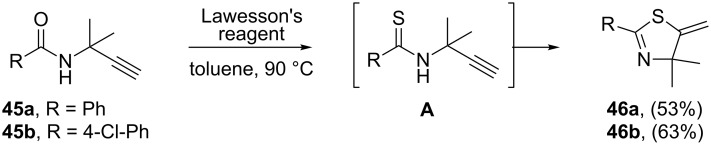
Synthesis of dihydrothiazoles **46** through the treatment of *N*-propargylamides **45** with Lawesson’s reagent.

Recently, Alhalib and Moran reported two examples for the preparation of fully substituted dihydrothiazoles **46** through the treatment of *N*-propargylamides **45** with Lawesson’s reagent in toluene. It is suggested that the *N*-(propargyl)thioamide intermediate **A** is initially formed, followed by a facile 5-*exo-dig* cyclization process to give the final products **46** in moderate yields (Scheme 12) [95].

**Scheme 13:**
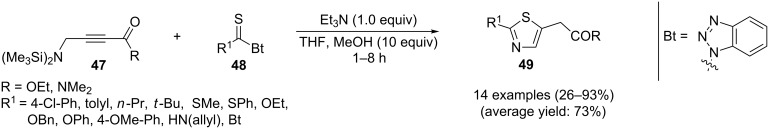
Synthesis of thiazoles **49** by treatment of silyl-protected *N*-propargylamines **47** with benzotriazolylthiones **48**.

**Scheme 14:**
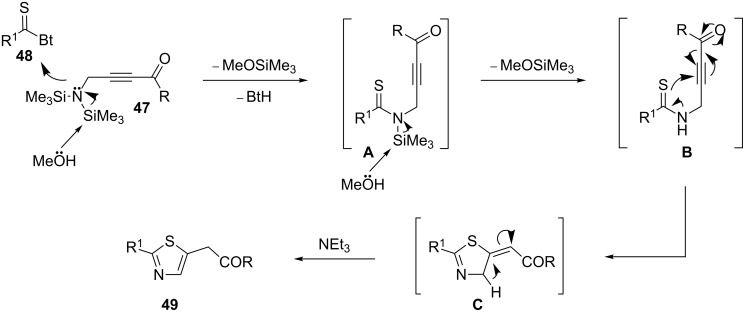
Mechanism proposed to explain the synthesis of 2,5-disubstituted thiazoles **49** developed by Sasmal.

An important study on 2,5-disubstituted thiazoles **49** was carried out by Sasmal, Sridhar, and Iqbal. The authors converted silyl-protected *N*-propargylamines **47** into thiazoles **49** by their treatment with benzotriazolylthiones **48** in a THF/MeOH/Et_3_N system (Scheme 13). The proposed mechanism for the reaction starts with the generation of the *N*-(propargyl)thioamide intermediates **A** through a thioacylation of *N*-propargylamine **47** with benzotriazolylthione **48**. Then *N*-desilylation of **A** furnishes intermediate **B** which undergoes a base-promoted cyclization to give the intermediate **C**. Finally, the isomerization of **C** affords the observed products **49** (Scheme 14) [96].

**Scheme 15:**
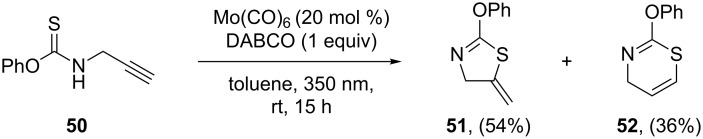
Mo-catalyzed cyclization of *N*-propargylthiocarbamate **50**.

In 2011, X. Meng and S. Kim reported an example of thiazolidine preparation through a Mo-catalyzed 5-*exo-dig* cyclization of the *N*-propargylthiocarbamate **50** in toluene under irradiation at 350 nm. As shown in Scheme 15 the target 2-phenoxy-substituted thiazolidine **51** was obtained in a yield of 54% along with the product originating from a 6-*endo-dig* cyclization [97].

**Scheme 16:**
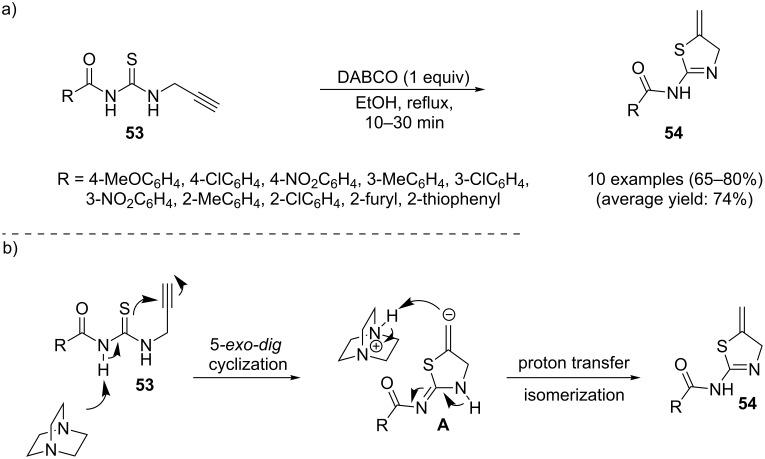
(a) DABCO-mediated intramolecular cyclization of *N*-(propargylcarbamothioyl)amides **53** to the corresponding dihydrothiazol-2-ylamides **54**. (b) Possible reaction pathway for the generation of product **54**.

Recently, Foroumadi and co-workers studied the possibility of synthesizing thiazole derivatives from *N*-propargylthioureas through a regioselective 5-*exo-dig* cyclization–proton transfer–isomerization sequential process. They found that the easily available *N-*(propargylcarbamothioyl)amides **53** in the presence of 1,4-diazabicyclo[2.2.2]octane (DABCO) as the base in refluxing ethanol, rapidly cyclized and produced the corresponding dihydrothiazol-2-ylamides **54** in good yields (Scheme 16a). The mechanism for this cyclization as proposed by the authors is depicted in Scheme 16b [98].

**Scheme 17:**
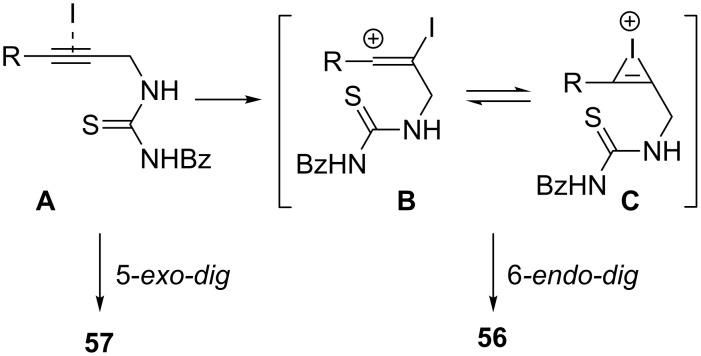
Proposed mechanism for the generation of the iodine-substituted 4*H*-1,3-thiazines **56** and 4,5-dihydrothiazoles **57**.

Following this work, the Čikotienė group studied the metal-free halogen, chalcogen, or oxocarbenium ion-mediated cyclization of a series of *N*-propargylthioureas **55** (Table 2). Some important information of the reactions are listed below: (1) iodine-mediated cyclizations of terminal *N*-propargylthioureas **55** gave exclusively 4,5-dihydrothiazoles **57** through a 5-*exo*-*dig* cyclization, whereas internal *N*-propargylthioureas **55** under the same reaction conditions gave a mixture of 4*H*-1,3-thiazines **56** and 4,5-dihydrothiazoles **57**. The mechanistic course of this reaction sequence is shown in Scheme 17 and involves the initial formation of the charge-transfer complex **A** between the iodonium ion and the triple bond. The 5-*exo-dig* cyclization of this intermediate gives rise to 4,5-dihydrothiazoles and the competing 6*-endo-dig* ring-closing process affords 4*H*-1,3-thiazines after conversion of the charge-transfer complex into the ring-opened iodovinyl **B** or bridged iodirenium **C** ions; (2) bromine-mediated cyclizations of both electron-poor and electron-rich *N*-propargylthioureas **55** gave exclusively 4,5-dihydrothiazoles **57** in moderate to good yields; (3) phenyl hypochloroselenoite-mediated cyclizations of terminal *N*-propargylthioureas **55** underwent a regioselective 5-*exo-dig* cyclization giving the corresponding 4,5-dihydrothiazoles **57** in moderate yields. On the other hand internal *N*-propargylamines **55** under the same reaction conditions gave a mixture of **57** and **56**; (4) arylideneoxonium ion-mediated cyclization of internal *N*-propargylamines **55** afforded exclusively the corresponding 4*H*-1,3-thiazines **56** in good yields. However, terminal *N*-propargylamines failed to participate in this reaction [99].

**Table 2:** Electrophile-mediated cyclization of *N*-propargylthioureas **55**.

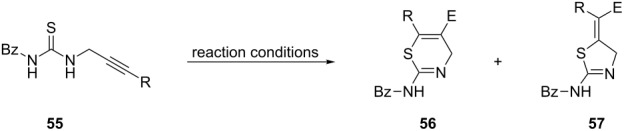					

Entry	R	Reaction conditions	E^+^	**56** Yield (%)	**57** Yield (%)

1	H	I_2_ (2 equiv), DCM, 0 °C	I^+^	–	20
2	Ph	I_2_ (2 equiv), DCM, 0 °C	I^+^	18	25
3	4-OMe-Ph	I_2_ (2 equiv), DCM, 0 °C	I^+^	59	traces
4	Ph	NBS (1.1 equiv), DCM, rt	Br^+^	–	67
5	4-OMe-Ph	NBS (1.1 equiv), DCM, rt	Br^+^	–	68
6	4-Cl-Ph	NBS (1.1 equiv), DCM, rt	Br^+^	–	44
7	H	PhSeCl (1 equiv), DCM, 0 °C	PhSe^+^	–	45
8	Ph	PhSeCl (1 equiv), DCM, 0 °C	PhSe^+^	56	traces
9	4-OMe-Ph	PhSeCl (1 equiv), DCM, 0 °C	PhSe^+^	73	–
10	4-Cl-Ph	PhSeCl (1 equiv), DCM, 0 °C	PhSe^+^	51	–
11	H	ArCH(OR^1^)_2_ (1.5 equiv), TMSOTf (1 equiv), DCM, −10 °C	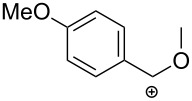	–	–
12	Ph	ArCH(OR^1^)_2_ (1.5 equiv), TMSOTf (1 equiv), DCM, −10 °C	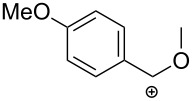	81	–
13	Ph	ArCH(OR^1^)_2_ (1.5 equiv), TMSOTf (1 equiv), DCM, −10 °C	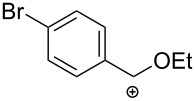	60	–
14	Ph	ArCH(OR^1^)_2_ (1.5 equiv), TMSOTf (1 equiv), DCM, −10 °C	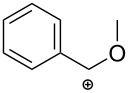	57	–
15	4-OMe-Ph	ArCH(OR^1^)_2_ (1.5 equiv), TMSOTf (1 equiv), DCM, −10 °C	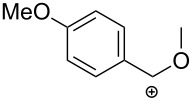	76	–
16	4-OMe-Ph	ArCH(OR^1^)_2_ (1.5 equiv), TMSOTf (1 equiv), DCM, −10 °C	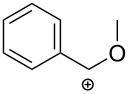	59	–
17	4-OMe-Ph	ArCH(OR^1^)_2_ (1.5 equiv), TMSOTf (1 equiv), DCM, −10 °C	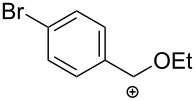	58	–
18	4-Cl-Ph	ArCH(OR^1^)_2_ (1.5 equiv), TMSOTf (1 equiv), DCM, −10 °C	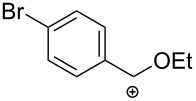	57	–
19	4-Cl-Ph	ArCH(OR^1^)_2_ (1.5 equiv), TMSOTf (1 equiv), DCM, −10 °C	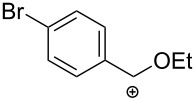	65	–

### 4 Miscellaneous

**Scheme 18:**
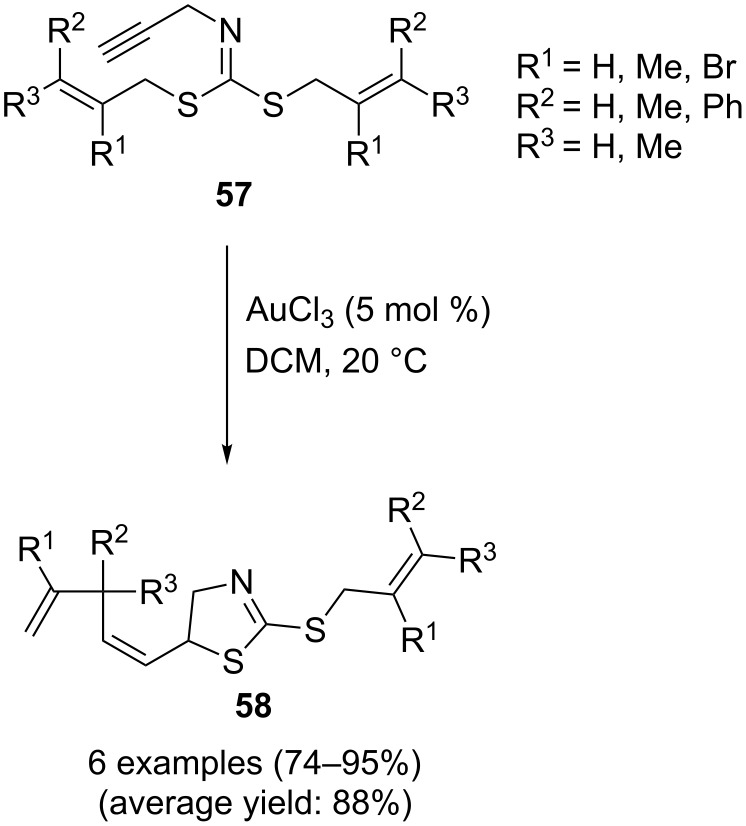
Au(III)-catalyzed synthesis of 5-alkylidenedihydrothiazoles **58** developed by Stevens.

Recently, Stevens and co-workers reported a robust protocol towards dihydrothiazoles through an Au(III)-catalyzed intramolecular cyclization of the corresponding dithiocarboimidates. Thus, the corresponding 5-alkylidene-dihydrothiazoles **58** were synthesized in good to excellent yields from *N-*(propargyldithiocarbo)imidates **57** through a 5-*exo-dig* cyclization followed by a thio-Claisen-type rearrangement with AuCl_3_ as the catalyst in dichloromethane (Scheme 18). It is worth mentioning that the required *N-*(propargyldithiocarbo)imidates were easily prepared in high yields through a condensation of commercially available and cheap *N*-propargylamine, allyl bromide, and carbon disulfide [100].

## Conclusion

Much work has been carried out during the past decade and has demonstrated that *N*-propargylamines are one of the most useful and versatile precursors in the synthesis of various nitrogen heterocycles and complex natural products. In this regard, recently an impressive increase in the number of publications on the preparation of thiazoles and their hydrogenated analogues through inter- and intramolecular cyclization of *N*-propargylamine derivatives appeared in the literature. In this review we discussed the most representative and interesting reports on this emerging field. As illustrated, the processes provided the title compounds in good yields with fewer steps and higher atom economy than previously reported examples. We hope that this review will encourage synthetic organic chemists to employ these valuable methodologies to the synthesis of important new thiazole derivatives.
